# Identification of suitable qPCR reference genes for the normalization of gene expression in the BL10-*mdx* and D2-*mdx* mouse models of Duchenne muscular dystrophy

**DOI:** 10.1371/journal.pone.0318944

**Published:** 2025-02-25

**Authors:** Kayleigh Putker, Anne-Fleur Schneider, Davy Van De Vijver, John Hildyard, Annemieke Aartsma-Rus, Maaike van Putten

**Affiliations:** 1 Department of Human Genetics, Leiden University Medical Center, Leiden, The Netherlands; 2 Department of Clinical Sciences and Services, Comparative Neuromuscular Diseases Laboratory, Royal Veterinary College, London, United Kingdom; Fujita Health University, JAPAN

## Abstract

Duchenne muscular dystrophy (DMD) is an X-linked disorder that is caused by mutations in the *DMD* gene, leading to progressive muscle wasting and weakness. There is currently no cure for DMD. The BL10-*mdx* mouse is the most commonly used model in preclinical DMD studies, but it exhibits a mild disease phenotype compared to DMD patients, limiting research translatability. The newer D2-*mdx* mouse has a more severe phenotype at an early age and may better recapitulate human disease. To compare these mouse models on a transcriptional level with quantitative RT-PCR, stable and reliable reference genes are indispensable. We aimed to evaluate the stability and reliability of a panel of nine candidate reference genes (*Actb, Ap3d1, Gapdh, Hmbs, Htatsf1, Pak1ip1, Rpl13a, Sdha* and *Zfp91*) in the gastrocnemius, diaphragm and heart of mice from both strains and their corresponding wild types aged 4 to 52 weeks. Data was analyzed using geNorm, BestKeeper, deltaCt and NormFinder. We found that *Htatsf1, Pak1ip1* and *Zfp91* are suitable reference genes for normalization of gene expression in dystrophic and healthy mice, regardless of the tissue type or age. In our hands, *Actb, Gapdh* and *Rpl13a* were not suitable as reference genes, exhibiting tissue-, age-, or disease specific changes in expression. This study highlights the importance of the selection of suitable reference genes, as their stability can differ between specific experimental setups.

## Introduction

Duchenne muscular dystrophy (DMD) is a muscle wasting neuromuscular disorder affecting 1 in 5000 newborn males [1]. It is caused by mutations in the *DMD* gene, resulting in the absence of functional dystrophin protein [2]. Dystrophin is an important component of the dystrophin glycoprotein complex, which connects the actin cytoskeleton to the extracellular matrix of muscle fibers and provides strength and stability [3,4]. In the absence of dystrophin, the dystrophin glycoprotein complex is disrupted, rendering muscle fibers highly susceptible to damage by mechanical forces during muscle contractions [5]. Chronic cycles of muscle degeneration and regeneration exhaust the muscle’s regenerative capacity, leading to progressive replacement of muscle tissue by connective and adipose tissue and consequent loss of muscle function [6,7]. In DMD patients, proximal muscle weakness is one of the first clinical symptoms that is observed between the age of 3-5 years. Patients become wheelchair dependent around the age of 12, and eventually die prematurely due to respiratory and cardiac failure [8,9]. There is no cure for DMD, but DMD patients are treated with corticosteroids as part of the standards of care to maintain muscle strength and prolong ambulation [10,11].

The C57BL/10ScSn-*Dmd*^*mdx*^/J (BL10-*mdx*) mouse is the most commonly used preclinical model to study potential therapeutics for DMD [12]. BL10-*mdx* mice harbor a premature stop codon mutation in exon 23 of the *Dmd* gene, which hampers the synthesis of dystrophin. From the age of 3 weeks onwards, BL10-*mdx* mice undergo cycles of muscle degeneration and regeneration, but largely recover from this through their excellent regenerative capacity [13]. Consequently, BL10-*mdx* mice live near-normal lifespans, and exhibit compensatory muscle hypertrophy rather than atrophy: muscle functionality is only slightly impaired, and muscle pathology is milder, with limited fibrosis and adiposis compared to DMD patients [14–16]. This mild phenotype can also be explained by differences in body size, peak muscle loading or length of the growth phase, but regardless of the mechanism, the failure of BL10-*mdx* mice to recapitulate the severity of human DMD limits their utility in preclinical testing. To overcome this, there is need for a more severely affected mouse model that more closely reflects the severity of the disease.

The D2.B10-*Dmd*^*mdx*^/J (D2*-mdx*) mouse, generated by crossing BL10-*mdx* mice onto the DBA/2J genetic background [12], has a more severe disease phenotype with more fibrotic tissue and fat infiltration, and impaired regenerative capacities [17,18]. Moreover, these mice exhibit remarkable muscle weakness compared to the BL10-*mdx* mouse model [19]. D2-*mdx* mice carry a polymorphism in the coding region of the latent TGF-β-binding protein 4 gene (*Ltbp4*), which normally regulates the release of TGF-β in the extracellular matrix. This leads to an increase in active TGF-β levels during inflammation [20], which is associated with impaired regenerative myogenesis, and increased SMAD signaling (regulating differential gene expression in muscle cell development) and fibrosis [18,21]. In DMD patients, *LTBP4* variations and elevated TGF-β levels are associated with the age at loss of ambulation [22]. The D2-*mdx* model also has a mutation in the *Anxa6* gene, which negatively affects muscle repair [23,24], and carries the *Dyscalc* locus, located in either the *Abcc6* or *Emp3* gene, which is associated with the accumulation of calcium deposits in skeletal and cardiac muscle following necrosis [25–27]. Given their pronounced dystrophic phenotype, the D2*-mdx* mouse may be of interest for preclinical analysis. Notably, the disease pathology is most pronounced in young mice and is largely resolved in 34-week-old mice [28]. Despite the increasing number of studies, a more complete natural history study, which directly compares aspects on muscle function, histology and pathology markers for the D2-*mdx* and BL10-*mdx* models at different timepoints is lacking [28,29]. To gain deeper insights into the disease progression of both mouse models, a detailed longitudinal study is ongoing using complementary approaches in an independent experimental effort together with Prof. Dr. Annamaria De Luca’s lab at the University of Bari (UniBa). In this collaborative effort we investigate function and pathology of skeletal muscles and the heart from D2-*mdx* and BL10-*mdx* mice and their corresponding wild types over time. Here, we focus on the identification of suitable reference genes to normalize gene expression data of known pathology biomarkers.

Normalization of gene expression data is an essential component of a reliable quantitative Real-Time Polymerase Chain Reaction (qRT-PCR) assay [30]. Due to the numerous sources of variability that can mask changes in gene expression, including sample input, RNA integrity, efficiency of cDNA synthesis, and differences in overall transcriptional activity, normalization is essential to ensure that measurements can reliably be compared between samples [31]. The usage of stable reference genes that are consistently expressed across all samples and conditions is the most common method for normalizing data. Stable reference genes can be determined a priori by assessing the expression stability of multiple potential candidates within a representative sample dataset. As stable gene expression can vary in a stochastic fashion, the use of a single reference gene for normalization is discouraged, and two or three stable candidates are recommended [32,33]. While healthy muscle exhibits remarkably stable transcriptional stability, dystrophic muscle shows a highly variable gene expression landscape due to the diverse range of cell types, making it challenging to select appropriate reference genes [34]. Acknowledgement of the biological role of candidate reference genes in the selection process is therefore of importance. Nevertheless, multiple reference genes appear suitable in the BL10-*mdx* mouse model, regardless of the tissue type or age. In our study, we assessed the suitability of a panel of nine candidate reference genes selected from previously published studies in the BL10-*mdx* mouse (*Actb, Ap3d1, Gapdh, Hmbs, Htatsf1, Pak1ip1, Rpl13a, Sdha* and *Zfp91*), including two of the most prevalently used reference genes (*Actb* and *Gapdh*) that have been shown to be poor references under some circumstances [35,36]. Our sample set consisted of tissues from dystrophic (BL10-*mdx* and D2-*mdx*) and healthy (BL10-wt and D2-wt) mice collected at the clinically-relevant ages of 4, 8, 12, 28 and 52 weeks. We tested the candidate reference genes in two skeletal muscles: the gastrocnemius, which is commonly used in studies involving *mdx* mice, and the diaphragm, which is more severely affected and thus more closely related to the muscle pathology in DMD patients. Additionally, we included the heart as DMD patients suffer from cardiomyopathy. Using multiple mathematical approaches (geNorm, NormFinder, BestKeeper, and deltaCt method), we were able to identify strong universal reference genes that can be used for normalization of gene expression data.

## Materials and methods

The animal experiments were approved by and performed following the guidelines of the Centrale Commissie Dierproeven (AVD1060020171407) and the Animal Welfare Body of the Leiden University (PE.17.246.022).

### Animals and tissue collection

C57BL/10ScSn-*Dmd*^*mdx*^/J (BL10-*mdx*), D2.B10-*Dmd*^*mdx*^/J (D2*-mdx*), C57BL/10ScSnJ (BL10-wt) and DBA/2J (D2-wt) male mice were bred in the Experimental Animal Facility of the Leiden University Medical Center. Breeding couples were obtained from Jackson Laboratory (USA, distributed by Charles River). Animals were housed in individually ventilated cages (2-5 mice per cage) with 12 hour light/dark cycles at 20.5°C and had *ad libitum* access to RM3 chow (Special Diets Services, Essex, UK) and water. Mice were randomized over six experimental groups, each consisting of 5 males per strain, that were sacrificed by cervical dislocation at either 4, 8, 12, 28 or 52 weeks of age. The gastrocnemius, diaphragm and heart were isolated and mounted with Tissue-Tek Optimal Cutting Temperature (O.C.T.) compound (Sakura Finetek, Torrance, CA, USA) on a piece of cork. Samples were snap frozen in liquid nitrogen cooled 2-methylbutane and stored at −80°C until further processing. The heart was only isolated from mice aged 8 weeks or older due to lack of pathology in younger mice.

### RNA isolation

Frozen tissues were cut in sections of 8 µm using a CM3050 S Cryostat (Leica Biosystems, Buffalo Grove, IL, USA) and mounted on SuperFrost Adhesion slides (Epredia, Kalamazoo, MI, USA). Intermediate sections were collected in 1.4 mm Zirconium Beads Pre-Filled Tubes (OPS Diagnostics, Lebanon, NJ, USA) for RNA isolation. Samples were homogenized in TRIsure isolation reagent (Bioline, London, United Kingdom) using a MagNA Lyser (Roche Diagnostics, Basel, Switzerland), and total RNA was isolated according to the manufacturer’s protocol. RNA was further purified by using the NucleoSpin RNA II kit (Macherey-Nagel, Düren, Germany) according to the manufacturer’s instructions. RNA purity and quantity were assessed via spectrometry using the NanoDrop ND-1000 Spectrophotometer (Thermo Fisher Scientific, Waltham, MA, USA). All 280 samples had a 260/280 ratio of > 2.0, and 221 of the 280 samples had a 260/230 ratio of > 1.7.

### Complementary DNA synthesis

Complementary DNA (cDNA) was synthesized from 300 ng total RNA by incubating each RNA sample with 1 µl random hexamer primers (40 ng/µl, Thermo Fisher Scientific) and 1 µl deoxynucleotide triphosphate mix (10 µM of each nucleotide, Invitrogen, Waltham, MA, USA) in a total volume of 10 µl at 70°C for 5 min, after which samples were put on ice. To each RNA sample, 1 µl of M-MLV Reverse Transcriptase (220 U/µl), 4 µl of M-MLV 5X Reaction Buffer, 0.5 µl of recombinant RNasin Ribonuclease Inhibitor (40 U/µl, Promega, Madison, WI, USA) and 4.5 µl nuclease-free water was added. Samples were incubated at room temperature for 10 min, followed by 42°C for 60 min with reaction termination at 70°C for 10 min. cDNA was diluted ten times with 180 µL nuclease-free water to prevent inhibition of downstream PCR, leading to a final cDNA concentration of ~ 1.5 ng/µL (assuming 1:1 conversion). Samples were stored at −20°C.

### Quantitative PCR (qPCR)

qPCR was performed using the LightCycler 480 Instrument II (Roche Diagnostics). Reactions were carried out in triplicate using 2 µL cDNA per well (approx. 3 ng cDNA), 4 µL 2x SensiMix SYBR Hi-ROX (Bioline) and 1 µL of both the forward and reverse primer (1 pmol/µL per primer) for a total volume of 8 µL per well. The polymerase was activated during a 10 min incubation step at 95°C, followed by 45 cycles of 95°C for 10 s, 60°C for 30 s and 72°C for 20 s. The expression levels were analyzed with the LinReg qPCR method [37], whereafter triplicates were averaged and converted to relative quantities (RQ). The primer pairs for the candidate reference genes *Actb, Ap3d1, Gapdh, Hmbs, Htatsf1, Pak1ip1, Rpl13a, Sdha* and *Zfp91* were designed in house using primer3 (primer3.ut.ee) (Table 1). Primers spanned exon/exon junctions and were selected based on their length (20 bp), melting temperature (59-61°C), size of the product (90-110 bp) and melting temperature of the product (80-85°C). Primer sequences were compared with the mouse reference (C57BL/6J) DNA database using BLAST (Ensembl). For each primer pair, amplification efficiency was evaluated using a dilution series of one cDNA template for both wild type strains.

**Table 1:** Primer sequences of the selected candidate reference genes and their full names and function.

Gene	Full name	Function	Direction	5’-3’ sequence
*Actb*	Beta Actin	Cell shape and motility	Forward	CCACCATGTACCCAGGCATT
			Reverse	GAGTACTTGCGCTCAGGAGG
*Ap3d1*	Adaptor-Related Protein Complex 3 Subunit Delta-1	Biogenesis and vesicle trafficking	Forward	CTGAAGCAGGACAACATCGC
			Reverse	ATGATGTTGAAGGCAGCCCA
*Gapdh*	Glyceraldehyde-3-Phosphate Dehydrogenase	Glycolysis pathway	Forward	TCCCACTCTTCCACCTTCGA
			Reverse	CCACCACCCTGTTGCTGTAG
*Hmbs*	Hydroxymethylbilane Synthase	Heme biosynthesis	Forward	CCCGTAGCAGTGCATACAGT
			Reverse	ATGGTGGCCTGCATAGTCTC
*Htatsf1*	HIV-1 Tat Specific Factor 1	Transcriptional elongation and splicing	Forward	TTTGGTGGCCGACAGATCAC
			Reverse	CAGCCGCTCCTCTCTTTCTC
*Pak1ip1*	PAK1 Interacting Protein 1	Cell proliferation and signal transduction	Forward	AAAGGAAGGTGGAGCATGGG
			Reverse	CGTCTTCTGCCCCACTGATT
*Rpl13a*	Ribosomal Protein L13	Protein synthesis	Forward	TCGTACGCTGTGAAGGCATC
			Reverse	TCGGGAGGGGTTGGTATTCA
*Sdha*	Succinate Dehydrogenase Complex, Subunit A, Flavoprotein (Fp)	Cellular energy production	Forward	CCAAGGACCTGGCATCAAGA
			Reverse	CGTGATCTTTCTCAGGGCCA
*Zfp91*	Zinc Finger Protein 91	Immune responses and autophagy	Forward	AGATGACAAAAGTCCGCGGT
			Reverse	CGAGGGTGAGCAAGAACAGT

### Data analysis

To rank the stability of the candidate reference genes, the dataset was analyzed using geNorm, BestKeeper, deltaCt and NormFinder. These methods use different approaches to determine the stability of the genes. Combining the results of these approaches increases the power of this study, with truly strong candidates consistently scoring high regardless of the method of assessment. BestKeeper and deltaCt use raw Cq values (average of triplicates), while both geNorm and NormFinder use linearized RQ values. Prior to using geNorm and NormFinder, Cq values were converted to RQ values by selecting the minimum Cq value per gene and subtracting this from all other values using the formula *Efficiency^(minimum Cq value – Cq value).* All methods were carried out in Microsoft Excel for Microsoft 365 [38].

#### geNorm.

With geNorm, the gene expression stability measure M was determined [39]. RQ values were used to analyze the average pairwise variation of each individual candidate gene with all other candidates using the geNorm Excel macro. The gene with the lowest correlation (thus the highest M value) was excluded, whereafter the process was repeated until only two highly correlated genes remained: the ‘best pair’. The M value of the best pair, together with the M values of each discarded gene, were ranked from least to most stable, where the genes of the best pair (with the lowest M value) were considered the most stably expressed genes of the dataset. Candidate reference genes with M values <  0.5 are generally considered as suitable within relatively homogeneous datasets, while an M value <  1.0 is typically accepted within more heterogeneous datasets with high transcriptional variability [40]. In addition, the optimal number of reference genes to be used for normalization in a given experiment was calculated by pairwise variation analysis. This is determined by V values, indicating to what extent adding an additional reference gene enhances normalization accuracy. The geNorm Excel macro is available on request.

#### BestKeeper.

Similar to geNorm, BestKeeper determined the most stable reference gene using a pairwise correlation analysis [41]. Here, the per-sample geometric mean of all candidates was calculated (the ‘BestKeeper index’), reflecting the average behavior of the entire dataset. Genes were ranked by correlation with the BestKeeper index (Pearson correlation: r), from least to most stable, where r = 1 indicates a perfect correlation. Bestkeeper is available as a prepared Excel spreadsheet for up to 10 genes and 100 samples (http://www.gene-quantification.de/bestkeeper.html). For this study a custom spreadsheet, suitable for up to 20 genes and 400 samples, was used (available on request).

#### deltaCt.

The deltaCt method calculates the relative per-sample expression difference in pairs of reference genes (*deltaCt* =*  Cq gene 1−Cq gene 2*), and then determines the standard deviation of all deltaCt values: genes with similar expression patterns should exhibit consistent deltaCt values regardless of absolute expression, and thus low standard deviation [42]. For each sample, the absolute difference between Cq values, the mean and standard deviation were calculated. Genes were ranked by the average standard deviation across all pairwise comparisons, where lower standard deviations indicate a higher stability ranking thus a more stable gene.

#### NormFinder.

With NormFinder, the stability value was calculated for all genes in our dataset [31]. The overall expression variation of the candidate reference genes, as well as the variation between subsets of the data, was estimated by a mathematical model of intra- and intergroup variability. The given stability value for each gene is a measure of expression variation, where lower stability values indicate a more stable gene, either in the whole dataset (ungrouped) or within and between given subsets (grouped). For both ungrouped and grouped analyses, RQ values were used in the Excel Add-in NormFinder to calculate the stability value. For grouped analyses, sample groups were marked by identifiers: a unique label was assigned to each sample group, enabling NormFinder to differentiate between different groups (presence of disease, tissue type, strain or age) and assessing both intergroup and intragroup variation. NormFinder is available as Excel plugin (https://www.moma.dk/software/normfinder).

#### Aggregate rankings.

The outputs of all approaches were used to generate an aggregate ranking within different datasets: the whole dataset, dataset as healthy or dystrophic samples alone, and tissue specific (gastrocnemius, diaphragm and heart). For geNorm, the genes of the ‘best pair’ were considered to be of equal rank, and therefore the same value was used for both genes. BestKeeper ranked candidate reference genes by their Pearson correlation coefficient, where high values represent a strong correlation, whereas the other approaches ranked genes by their stability values with high values representing low stability. To correct for this, values from BestKeeper were first inverted (1-value), whereafter all values were normalized to the highest value. Per gene, the geometric mean was calculated from the normalized BestKeeper values along with the values of all other approaches. Based on these results, three high scoring genes were used to generate a normalization factor (i.e., geometric mean of these three high scoring genes) for gene validation. Data from three low scoring genes was normalized using this normalization factor, and the mean per-group coefficient of variation (CoV) was calculated to assess the effectiveness of normalization, which is crucial for accurate and reliable gene expression analyses as this would correct for technical variability rather than biological differences. CoV values were obtained by calculating the average of the individual CoVs per time-point, per tissue. Raw RQ values, without normalization, were compared to RQ values normalized to the geometric mean of the three high scoring genes*.*

The geNorm, BestKeeper, deltaCt and NormFinder analyses were conducted on the whole dataset (*n* = 280 samples), and on different subsets:

- Disease-specific: healthy (*n* = 140) and dystrophic (*n* = 140)
- Tissue-specific: gastrocnemius (*n* = 100), diaphragm (*n* = 100) and heart (*n* = 80)
- Strain-specific: BL10-wt, D2-wt, BL10-*mdx* and D2-*mdx* (*n* = 70 per strain)
- Age-specific: 4 weeks (*n* = 40), and 8, 12, 28 and 52 weeks of age (*n* = 60 per age category)

## Results

### Cq values differed between genes, but were stable between samples

**Fig 1:**
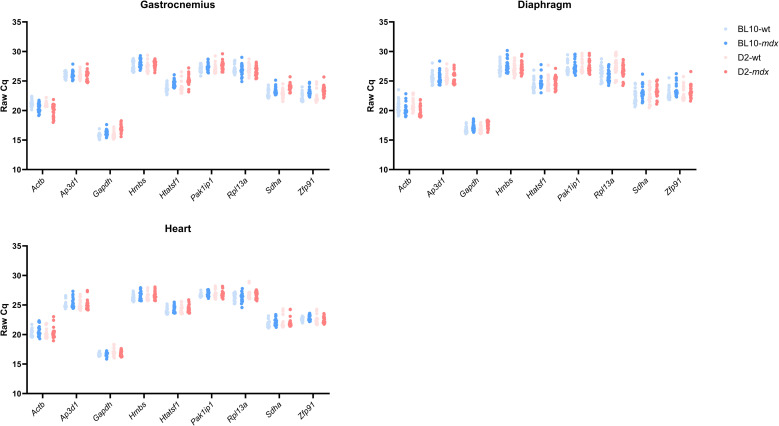
Raw quantification cycle (Cq) values. Cq values for each candidate reference gene in the gastrocnemius, diaphragm and heart. Each datapoint represents the mean Cq value of an individual sample for each gene. Data is separated by strain: Light blue: BL10-wt; Dark blue: BL10-*mdx*; Light red: D2-wt; Dark red: D2-*mdx*.

The expression level of all candidate reference genes was determined in 279 samples from 100 animals (Fig 1). One sample was excluded from further analysis due to failed cDNA synthesis (gastrocnemius, D2-*mdx*, 28 weeks of age). Cq values were consistent with expected transcript abundance: highly expressed mRNAs, *Gapdh* and *Actb*, showed lowest mean Cq (16.7 and 20.5 respectively). Mean Cq values for other genes encompassed a range from 22.8-27.2. Typically, Cq values between 15 and 28 are considered appropriate, thus all genes were considered suitable for this study. Minimal variability between strains was observed, and Cq values were also largely comparable between tissues (S1 Table). The distribution of Cq values within the whole dataset, regardless of the tissue type or strain, was lowest for *Gapdh* (min-max value: 15.1-18.6), *Pak1ip1* (25.8-29.7) and *Ap3d1* (24.1-28.4), and highest for *Sdha* (20.5-26.2) (S2 Table).

### geNorm consistently identified *Htatsf1* and *Zfp91* as the best pair

**Fig 2:**
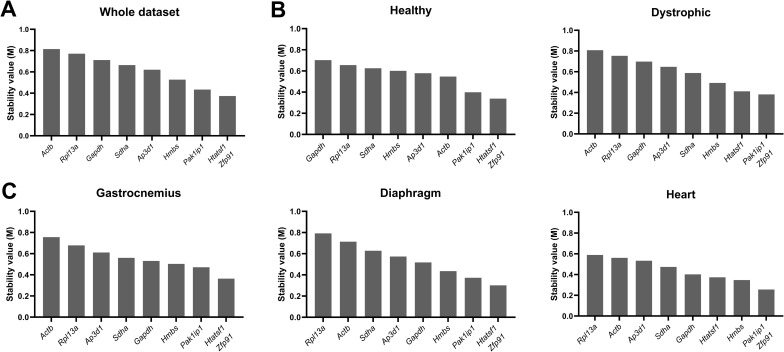
geNorm rankings. Rankings of the pairwise stability value M of the nine candidate reference genes, assessed by the geNorm method, for (**A**) the whole dataset, (**B**) healthy and dystrophic subsets, and (**C**) tissue-specific subsets. Genes are ranked from low stability (high M value) to high stability (low M value), where M values <  0.5 indicate highly stable genes. The highest scoring genes (the ‘best pair’) are considered to be equal in M value.

With the pairwise approach of geNorm, the stability measure M was determined. Candidate reference genes with M values <  0.5 are generally considered as suitable reference genes in relatively homogeneous datasets. We first analyzed the entire dataset, regardless of the tissue or strain. Of the nine genes, only three were below 0.5, but this was expected due to the broad variety of tissues, strains and genotypes (Fig 2A). We identified *Htatsf1* and *Zfp91* as the best pair, with *Pak1ip1* ranking third. *Gapdh, Rpl13a* and *Actb* were the worst performing genes of the entire dataset, with M values of 0.7-0.8. Splitting the dataset into healthy and dystrophic subsets did not substantially alter rankings of the highest ranked genes (Fig 2B). *Htatsf1*, *Zfp91* and *Pak1ip1* were again the most favorable genes. *Rpl13a* and *Gapdh* performed poorly in both subsets, while *Actb* scored low in the dystrophic subset, but comparatively high in healthy. Assessment of tissue-specific subsets also did not substantially alter the data: *Htatsf1*, *Zfp91* and *Pak1ip1* remained high scoring in all three tissues, while *Actb* and *Rpl13a* scored poorly. M values in the heart-specific subset were lower than in either skeletal muscle (Fig 2C), suggesting that the heart might exhibit generally high transcriptional stability compared to skeletal muscles. The overall stability pattern for the gastrocnemius and diaphragm was comparable to the whole dataset and the dystrophic samples alone. Similar to the entire dataset, *Htatsf1, Zfp91* and *Pak1ip1* were identified as the best pair. *Rpl13a* and *Actb* were the lowest scoring genes. Interestingly, *Gapdh* performed slightly better in the tissue-specific subsets, with an M value below 0.5 in the heart (and just above in the gastrocnemius and diaphragm).

Analysis of strain-specific subsets consistently identified *Zfp91* alternately paired with *Htatsf1* or *Pak1ip1* as best pair, with the notable exception of the BL10-wt strain, where *Actb* and *Ap3d1* was considered the best pair (S1A Fig). *Actb* was ranked comparatively highly in the other wild type strain (D2-wt) as well. *Zfp91* and *Htatsf1* (ranked highly in the other subsets) were two of the least stable genes in the BL10-wt strain. However, assessment of samples in age-specific subsets again identified *Htatsf1, Zfp91* and *Pak1ip1* as the strongest candidates, with the former two forming the best pair in all ages except 4 weeks (S1B Fig). *Gapdh, Rpl13a* and *Actb* were consistently identified as less stable genes.

In addition to ranking candidate reference genes by their M value, geNorm also calculated the change in pairwise variation that results from inclusion of additional reference genes. Values of <  0.2 are typically considered acceptable [39] and for our data this threshold was consistently achieved by use of the best pair alone (S3 Table).

### BestKeeper considered *Pak1ip1* and *Zfp91* as suitable reference genes

**Fig 3:**
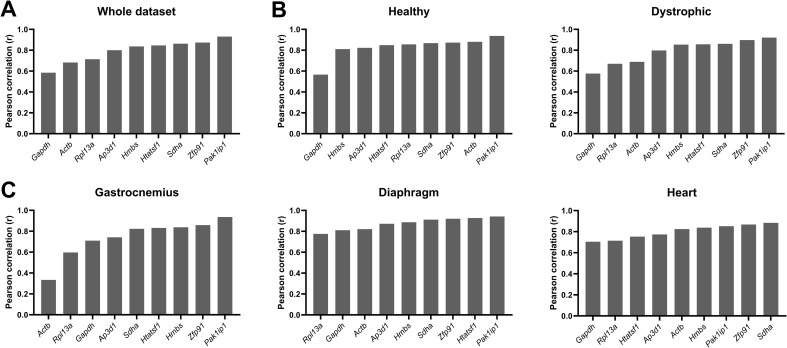
BestKeeper rankings. Rankings of the coefficient of correlation (r) of the nine candidate reference genes, assessed by the BestKeeper method, for (**A**) the whole dataset, (**B**) healthy and dystrophic subsets, and (**C**) tissue-specific subsets. Genes are ranked from low stability (low r) to high stability (high r).

Using pairwise comparison of each gene to the geometric mean of all candidate reference genes, genes were ranked by their Pearson correlation coefficient (r), with r =  1 indicating a perfect correlation. Overall correlations were high, with most genes achieving r values >  0.8, when assessed across the entire dataset (Fig 3A), and in healthy/dystrophic or tissue-specific subsets (Fig 3B–C). *Pak1ip1* and *Zfp91* were consistently ranked highly, which is in line with the geNorm analysis. The rankings of *Htatsf1* were less consistent, but typically only under comparisons where all scores were similar, such as in the heart-specific subset (Fig 3C). *Sdha* scored highly under most comparisons. In agreement with geNorm, *Rpl13a, Actb* and *Gapdh* typically scored poorly, though again *Actb* performed well in healthy samples alone, while it was one of the lowest scoring genes in the dystrophic subset. *Gapdh* was frequently ranked last, and exhibited a markedly lower correlation than other genes, with the notable exception of the gastrocnemius where *Rpl13a, Actb* and *Gapdh* were poorly correlated. In the tissue-specific subsets, r values suggested a more variable expression within gastrocnemius than within diaphragm or heart. These patterns were largely mirrored in strain- or age-specific subsets, with *Zfp91* and *Pak1ip1* scoring highly overall, usually accompanied by *Htatsf1* (S2A–B Fig)*. Gapdh* was universally ranked last. Correlation values in both wild type strains were higher than in either BL10-*mdx* or D2-*mdx*, and again *Actb* scored well in the healthy strains but not in dystrophic strains, nor in any age-specific subset. Stability in age-specific subsets suggested disease- or growth-associated variability, with the 8 weeks cohort being notably less stable than other age-specific subsets, which may be explained by the acute phase of pathology at this age. To combine the results of all age-specific subsets, the geometric mean rankings of all age-specific subsets was calculated, which produced rankings almost identical to those determined from the dataset assessed as a whole.

### *Pak1ip1* was consistently ranked highest in deltaCt analysis

**Fig 4:**
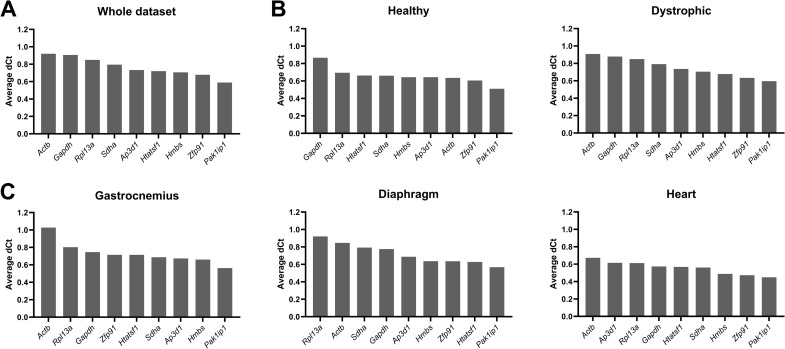
deltaCt rankings. Rankings of the average deltaCt standard deviation of the nine candidate reference genes, assessed by the deltaCt method, for (**A**) the whole dataset, (**B**) healthy and dystrophic subsets, and (**C**) tissue-specific subsets. Genes are ranked from low stability (high deltaCt score) to high stability (low deltaCt score).

In deltaCt analysis, all possible gene combinations are compared and the standard deviation of each comparison is calculated, ranking genes by mean standard deviation accordingly, with lower values indicating a higher stability. The ranking of the genes in the entire dataset or when subsampled by healthy and dystrophic samples alone was quite comparable (Fig 4A–B). The healthy sample subset in particular exhibited only modest differences between all candidate reference genes except the low ranking *Gapdh* (standard deviation of 0.87). *Pak1ip1* and *Zfp91* were the best performing genes in the three datasets, while *Rpl13a* and *Gapdh* were ranked poorest with standard deviations close to 1.0. *Actb* again showed a high variability, being the worst performing gene in the whole and dystrophic datasets, while being one of the best performing genes in the healthy subset. In the tissue-specific subsets, *Pak1ip1* was ranked highest, while *Zfp91* was similarly high scoring in all subsets except for the gastrocnemius. *Actb* was consistently ranked as one of the lowest scoring genes, together with *Rpl13a* and *Gapdh* (Fig 4C). The overall stability in the heart was notably high, with even the lowest ranked genes demonstrating only modest standard deviations. Results from the strain-specific subsets were similar to healthy and dystrophic subsets alone, with slight increases in stability for the wild type strains (S3 Fig). *Pak1ip1* and *Zfp91* ranked highest in each of the specific strains. Interestingly, *Hmbs* was ranked quite high (third) in the D2-wt and D2-*mdx* strains, while it was ranked low in the BL10-wt and BL10-*mdx* strains (eighth and sixth, respectively).

### Overall stability of all genes was relatively high in the NormFinder analysis

**Fig 5:**
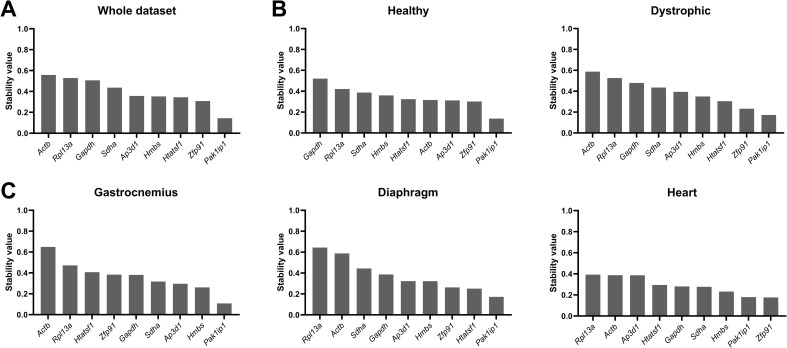
NormFinder rankings (ungrouped). Ungrouped rankings of the stability value of the nine candidate reference genes, assessed by the NormFinder method, for (**A**) the whole dataset, (**B**) healthy and dystrophic subsets, and (**C**) tissue-specific subsets. Genes are ranked from low stability (high stability value) to high stability (low stability value).

The NormFinder method uses relative quantities to assess the stability value for each gene, where a lower stability value indicates a higher suitability of the candidate reference gene. Using this approach stability values are calculated individually, rather than comparatively. Using an ungrouped approach, in which the entire dataset was analyzed without distinction between strains and tissues, NormFinder identified *Pak1ip1, Zfp91* and *Htatsf1* as the best genes in the assessment of the entire dataset, while *Actb, Rpl13a* and *Gapdh* scored poorly (Fig 5A). *Hmbs, Ap3d1* and *Sdha* were of modest stability. A similar pattern was observed in the specific subsets considering healthy or dystrophic samples (Fig 5B), with the exception of *Actb* that was again ranked as one of the best genes in the healthy subset only. Analysis of tissue-specific subsets also ranked *Pak1ip1* as best gene, and *Actb* and *Rpl13a* as least appropriate reference genes (Fig 5C). Although *Htatsf1* and *Zfp91* ranked highest in the entire dataset, in tissue-specific subsets ranking varied between tissue types. *Htatsf1* ranked low in the gastrocnemius and heart, and high in diaphragm, while *Zfp91* ranked low in the gastrocnemius it was found to be a good reference gene in diaphragm and heart. Stability in all tissues was overall high, with the heart showing particularly strong stability as all genes had stability values below 0.4, with *Zfp91* and *Pak1ip1* even below 0.25. Assessment of strain-specific subsets again placed *Pak1ip1*, *Zfp91* and *Htatsf1* highest, while *Rpl13a* and *Gapdh* were poor scoring (S4 Fig). *Pak1ip1* was consistently ranked highest in all strains. In the BL10-wt strain specifically (as with other methods, above) *Actb* was one of the top scoring genes.

**Fig 6:**
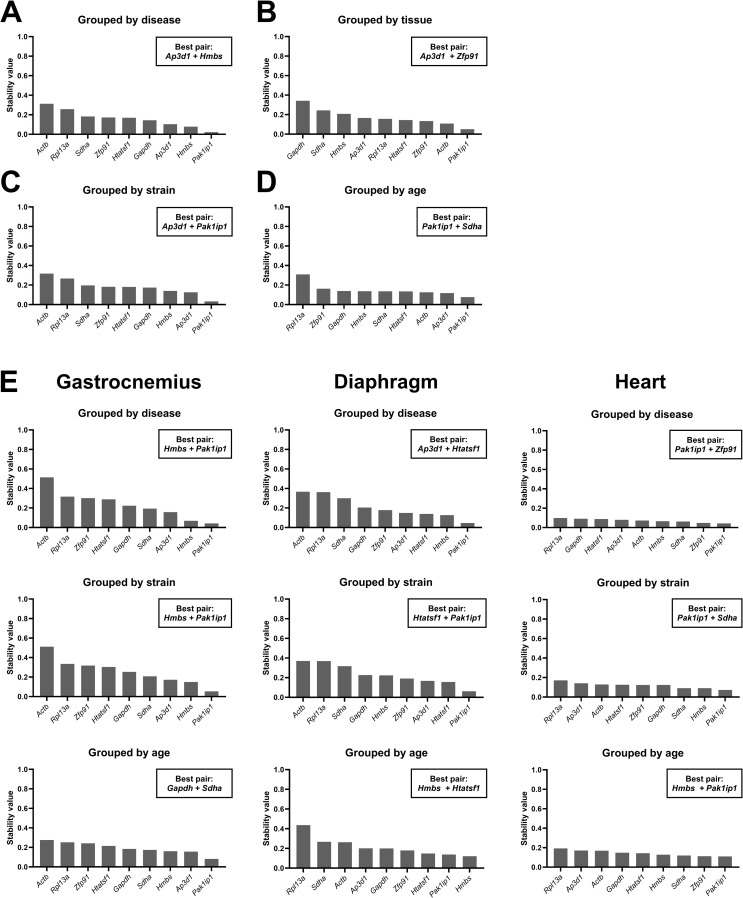
NormFinder rankings (grouped). Grouped rankings of the stability value of the nine candidate reference genes, assessed by the NormFinder method. The whole dataset was grouped by (**A**) disease, (**B**) tissue, (**C**) strain, or (**D**) age. For the gastrocnemius, diaphragm and heart samples, genes were grouped by (**E**) disease, strain, or age. Genes are ranked from low to high stability (high to low values). For each grouping, the best pair is indicated on the plot.

Data was also analyzed by grouped analysis, in which the entire dataset was grouped by presence of disease, tissue type, strain or age, and tissue-specific subsets were grouped by presence of disease, strain or age. Following grouped analysis a best pair was determined, two genes that confer greater stability together than as individual genes alone. Overall, the best pair did not necessarily consist of the most stable genes of that group. Interestingly, for almost all grouped analyses, stability values were <  0.4 (Fig 6), suggesting that the bulk of our candidate reference gene panel is not substantially affected by disease, tissue type, strain or age, with *Actb* being one exception, scoring markedly more poorly in all comparisons grouped by disease or strain, suggesting – as demonstrated by other approaches – that this gene is highly disease-sensitive. Specific ranking of individual genes differed slightly between groupings, but *Pak1ip1* was overall the most stable gene. *Ap3d1*, one of the genes with modest stability in previous methods, was ranked highly when the entire dataset was grouped by either disease, tissue type, strain or age, and was even part of the best pair when grouped by disease, tissue type or strain.

Apart from the overall low stability values, most genes were ranked high in the ungrouped and grouped NormFinder analysis, except for *Rpl13a* and *Actb* that consistently scored low. In most cases, the best pair did not consist of the highest ranked gene, but this was expected as most genes scored below 0.4.

### Aggregate rankings identified *Htatsf1, Pak1ip1* and *Zfp91* as suitable reference genes

**Fig 7:**
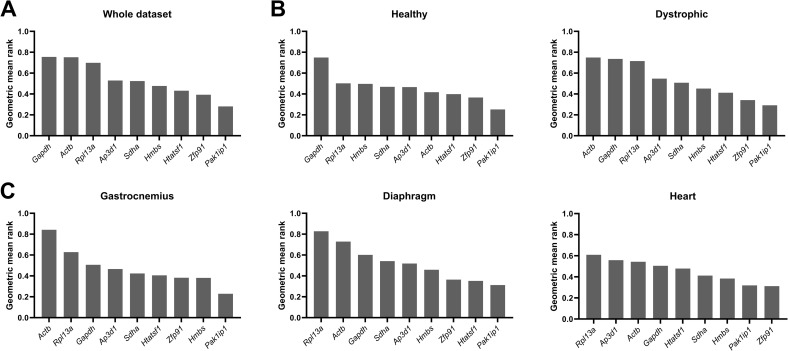
Aggregate rankings of the four methods. Rankings of the geometric mean scores of the nine candidate reference genes from all four analysis methods, for (**A**) the whole dataset, (**B**) healthy and dystrophic subsets, and (**C**) tissue-specific subsets. Genes are ranked from low stability (high geometric mean rank) to high stability (low geometric mean rank).

To obtain a comprehensive and balanced ranking of the studied candidate reference genes, we integrated the results of all four methods and calculated the geometric mean of the stability values derived from these methods. *Rpl13a, Actb* and *Gapdh* were near-universally ranked last in all subsets, suggesting that these genes are not suitable as reference genes in the analyzed models and tissue types (Fig 7). *Htatsf1, Pak1ip1* and *Zfp91* were found to be the strongest genes, regardless of the presence of a dystrophic genotype (Fig 7A–B). This suggests that these genes are suitable candidates, which was confirmed in the analysis per tissue type (Fig 7C). *Htatsf1, Pak1ip1* and *Zfp91* were considered strong in the gastrocnemius and diaphragm. In heart, *Pak1ip1* and *Zfp91* remained the strongest genes, followed by *Hmbs* and *Sdha,* with *Htatsf1* being ranked fifth. However, overall stability was relatively high in heart, with only a slight change in geometric means. *Pak1ip1* was considered to be the strongest gene of the panel, being ranked first or second (in heart) in all consensus scores.

The ranking of the candidate reference genes obtained from each of the methods previously described is summarized in Table 2.

**Table 2:** Overview of the candidate reference genes and their ranking for each analysis method.

			*Actb*	*Ap3d1*	*Gapdh*	*Hmbs2*	*Htatsf1*	*Pak1ip1*	*Rpl13a*	*Sdha*	*Zfp91*
**geNorm**	**Whole dataset**		0.815	0.620	0.711	0.527	0.374	0.434	0.771	0.664	0.374
	**Disease**	Dystrophic	0.808	0.647	0.698	0.492	0.411	0.381	0.754	0.588	0.381
		Healthy	0.547	0.579	0.702	0.601	0.339	0.399	0.655	0.625	0.339
	**Tissue**	Gastrocnemius	0.756	0.611	0.531	0.503	0.364	0.471	0.678	0.560	0.364
		Diaphragm	0.714	0.574	0.518	0.435	0.301	0.373	0.792	0.628	0.301
		Heart	0.561	0.533	0.402	0.347	0.374	0.255	0.589	0.474	0.255
	**Strain**	BL10-wt	0.369	0.369	0.697	0.576	0.627	0.540	0.478	0.390	0.645
		BL10-*mdx*	0.635	0.596	0.678	0.457	0.302	0.355	0.724	0.539	0.302
		D2-wt	0.508	0.541	0.689	0.568	0.309	0.385	0.644	0.601	0.309
		D2-*mdx*	0.847	0.706	0.576	0.490	0.442	0.384	0.761	0.655	0.384
	**Age**	4 weeks	0.877	0.669	0.750	0.541	0.401	0.361	0.700	0.616	0.361
		8 weeks	0.808	0.652	0.739	0.504	0.317	0.427	0.691	0.585	0.317
		12 weeks	0.734	0.601	0.767	0.520	0.371	0.437	0.700	0.645	0.371
		28 weeks	0.657	0.571	0.746	0.493	0.294	0.386	0.701	0.610	0.294
		52 weeks	0.653	0.635	0.685	0.515	0.427	0.454	0.669	0.598	0.427
**BestKeeper**	**Whole dataset**		0.682	0.800	0.585	0.836	0.846	0.930	0.714	0.863	0.874
	**Disease**	Dystrophic	0.689	0.798	0.576	0.854	0.857	0.921	0.670	0.861	0.897
		Healthy	0.880	0.823	0.566	0.810	0.849	0.937	0.856	0.868	0.873
	**Tissue**	Gastrocnemius	0.334	0.742	0.710	0.838	0.831	0.936	0.597	0.823	0.859
		Diaphragm	0.821	0.872	0.810	0.887	0.927	0.942	0.775	0.912	0.920
		Heart	0.825	0.774	0.705	0.838	0.753	0.852	0.714	0.884	0.868
	**Strain**	BL10-wt	0.872	0.801	0.398	0.816	0.783	0.911	0.850	0.834	0.793
		BL10-*mdx*	0.777	0.818	0.571	0.860	0.869	0.909	0.695	0.886	0.911
		D2-wt	0.895	0.855	0.663	0.830	0.886	0.952	0.867	0.892	0.924
		D2-*mdx*	0.667	0.797	0.605	0.880	0.849	0.930	0.653	0.845	0.895
	**Age**	4 weeks	0.641	0.858	0.602	0.846	0.881	0.921	0.837	0.890	0.941
		8 weeks	0.556	0.717	0.396	0.845	0.781	0.911	0.748	0.813	0.839
		12 weeks	0.620	0.673	0.545	0.739	0.824	0.923	0.714	0.765	0.829
		28 weeks	0.813	0.897	0.703	0.899	0.930	0.965	0.771	0.906	0.954
		52 weeks	0.732	0.768	0.639	0.883	0.763	0.931	0.674	0.896	0.831
**deltaCt**	**Whole dataset**		0.919	0.732	0.905	0.706	0.720	0.589	0.849	0.794	0.678
	**Disease**	Dystrophic	0.908	0.736	0.878	0.704	0.677	0.595	0.849	0.791	0.633
		Healthy	0.635	0.643	0.866	0.643	0.662	0.510	0.695	0.660	0.605
	**Tissue**	Gastrocnemius	1.027	0.673	0.746	0.660	0.714	0.564	0.802	0.688	0.715
		Diaphragm	0.846	0.687	0.775	0.636	0.627	0.568	0.920	0.792	0.635
		Heart	0.672	0.615	0.574	0.488	0.569	0.449	0.612	0.561	0.474
	**Strain**	BL10-wt	0.647	0.647	0.882	0.670	0.665	0.527	0.659	0.653	0.616
		BL10-*mdx*	0.768	0.656	0.840	0.694	0.621	0.565	0.815	0.693	0.574
		D2-wt	0.613	0.622	0.848	0.609	0.651	0.494	0.699	0.659	0.581
		D2-*mdx*	0.961	0.779	0.858	0.670	0.697	0.604	0.842	0.850	0.661
	**Age**	4 weeks	0.993	0.678	0.951	0.751	0.675	0.621	0.759	0.826	0.685
		8 weeks	1.049	0.779	0.941	0.678	0.711	0.582	0.739	0.820	0.656
		12 weeks	0.876	0.720	0.880	0.665	0.682	0.543	0.748	0.789	0.658
		28 weeks	0.806	0.651	0.902	0.659	0.711	0.558	0.787	0.730	0.619
		52 weeks	2.301	1.685	3.930	2.029	1.316	1.440	1.523	1.325	1.240
**NormFinder (ungrouped)**	**Whole dataset**		0.558	0.357	0.506	0.352	0.344	0.144	0.528	0.436	0.308
	**Disease**	Dystrophic	0.587	0.394	0.479	0.350	0.305	0.172	0.526	0.435	0.232
		Healthy	0.316	0.311	0.520	0.360	0.324	0.137	0.421	0.387	0.301
	**Tissue**	Gastrocnemius	0.649	0.296	0.381	0.261	0.407	0.108	0.472	0.317	0.384
		Diaphragm	0.588	0.323	0.386	0.322	0.251	0.172	0.644	0.444	0.263
		Heart	0.387	0.386	0.280	0.232	0.295	0.180	0.393	0.277	0.176
	**Strain**	BL10-wt	0.308	0.320	0.540	0.360	0.339	0.156	0.353	0.394	0.327
		BL10-*mdx*	0.404	0.356	0.480	0.368	0.284	0.204	0.511	0.348	0.208
		D2-wt	0.323	0.295	0.503	0.345	0.312	0.118	0.447	0.383	0.267
		D2-*mdx*	0.711	0.415	0.447	0.290	0.320	0.142	0.525	0.503	0.251
	**Age**	4 weeks	0.844	0.315	0.534	0.430	0.337	0.162	0.425	0.455	0.232
		8 weeks	0.644	0.387	0.551	0.316	0.324	0.150	0.397	0.456	0.275
		12 weeks	0.493	0.363	0.510	0.334	0.307	0.104	0.440	0.453	0.319
		28 weeks	0.432	0.307	0.522	0.319	0.363	0.126	0.478	0.401	0.277
		52 weeks	0.398	0.370	0.409	0.311	0.338	0.164	0.374	0.382	0.274

Values represent the calculated stability values of the method used (geNorm, BestKeeper, deltaCt and NormFinder (ungrouped)) in the whole dataset and in all subsets (disease-, tissue-, strain- and age-specific). Green indicates a high ranking of the gene within the given dataset, red indicates a low ranking of the gene.

To evaluate the validity of the high-scoring candidate reference genes, we employed a within-dataset strategy that was used previously [34]. Using the three high-scoring genes *Htatsf1, Pak1ip1* and *Zfp91,* we normalized the expression of a selection of low scoring genes from our gene panel: *Actb, Gapdh* and *Rpl13a*.

**Fig 8:**
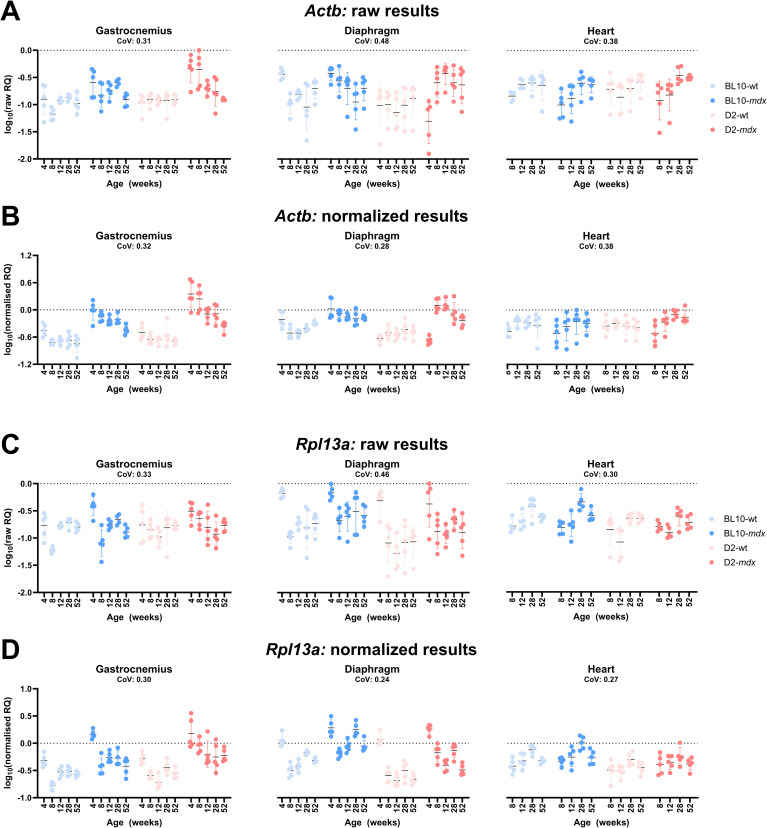
Normalization of *Actb* and *Rpl13a.* (**A**) Mean raw RQ values for *Actb* in gastrocnemius, diaphragm and heart. (**B**) Normalization to the geometric mean of *Htatsf1*, *Pak1ip1* and *Zfp91* in gastrocnemius, diaphragm and heart shows reduced variation. (**C**) Mean raw RQ values for *Rpl13a* in gastrocnemius, diaphragm and heart. (**D**) Normalization to the geometric mean of *Htatsf1, Pak1ip1* and *Zfp91* in gastrocnemius, diaphragm and heart shows reduced variation*.* Data is shown as log10 of RQ values. CoV values represent the average of the individual CoVs per time-point, per tissue. Data is separated by strain: Light blue: BL10-wt; Dark blue: BL10-*mdx*; Light red: D2-wt; Dark red: D2-*mdx*.

Raw RQ values for *Actb* (Fig 8A), the gene with the most variability in the previously described methods, suggested an association with strain, being higher expressed in the dystrophic strains compared to the healthy strains in the gastrocnemius and diaphragm, which was even more prominent after normalization to the geometric mean of *Htatsf1, Pak1ip1* and *Zfp91* (Fig 8B). Interestingly, normalized expression of *Actb* in healthy tissue was highly stable (explaining the higher scores of this gene in comparisons of the healthy strains) but exhibited a consistent decrease with increasing age in both dystrophic strains. Overall spread of data (coefficient of variation, CoV) was either reduced or unchanged following normalization. Normalization of *Rpl13a* revealed prominent age-associated changes: expression in gastrocnemius and diaphragm at 4 weeks was markedly higher than at subsequent time points (in both healthy and dystrophic strains), while overall expression was higher in dystrophic skeletal muscle specifically (Fig 8C–D).

*Gapdh* normalized to the geometric mean of *Htatsf1, Pak1ip1* and *Zfp91* showed a tissue-specific expression, being more abundant in the gastrocnemius, where it also exhibited a modest age-associated decline. Expression was moreover highly variable between samples, even within a group (S5A–B Fig). We also normalized raw RQ values of *Htatsf1* (S5C Fig) to the geometric mean of *Pak1ip1* and *Zfp91* (S5D Fig). Following normalization, the mean CoV markedly reduced in diaphragm (0.50 to 0.15), gastrocnemius (0.35 to 0.18) and heart (0.29 to 0.19), indicating that the normalization was effective. This was evident when comparing the raw RQ values and the normalized values, as overall variation was reduced following normalization. There was no association with age, disease, or tissue type visible.

Finally, we compared the normalization factor of the three high-scoring genes (*Htatsf1, Pak1ip1* and *Zfp91*) to the normalization factor of two of these genes. The normalization factor derived from the three high-scoring genes was nearly identical to that of *Pak1ip1* with either *Htatsf1* (S6A Fig) or *Zfp91* (S6B Fig), with gradients of 0.95 and 1.02 respectively, and Pearson correlations of 0.98. This suggests that utilization of two genes, and not three genes, would be sufficient for normalization of gene expression data.

## Discussion

The selection of appropriate reference genes is essential for quantitative gene expression studies, ensuring accurate normalization of gene expression levels and reliable results. In this study, we aimed to identify and validate candidate reference genes across various tissues and life stages of the BL10-*mdx* and D2-*mdx* mouse. By employing multiple methods, we identified a set of reliable reference genes for the normalization of gene expression data in these models.

In previous studies, *Pak1ip1* was one of the strongest genes in the developing mouse embryo [40], but was of modest stability in the BL10-*mdx* mouse model [34]. According to our results, *Pak1ip1* is a suitable reference gene for normalization of skeletal muscle and heart samples, regardless of the strain, tissue, or age. *Pak1ip1* was ranked highly in all analyses performed, both within the whole dataset and in different subsets. It did not exhibit any disease-, tissue- or age-specific changes in expression, suggesting that this gene is highly stable under all circumstances. Based on our results, *Htatsf1* and *Zfp91* appear to be strong reference genes as well, being ranked highly in most analyses. In line, the study of Hildyard *et al*. also showed that *Htatsf1* exhibits marked stability in cardiac samples of *mdx* mice [34]. *Zfp91* was also in the candidate panel of that study, but was omitted from subsequent analysis due to high variation in expression, something that we did not observe in our study. The methods used here are not identical to those used previously, however, and these discrepancies potentially highlight that along with strain, tissue type, age and disease state, even specific methodology can also contribute to variation. In addition, 59 out of 280 samples exhibited a 260/230 ratio below 1.7, indicating the presence of contaminants that absorb at 230 nm. Although RNA of all samples was purified over a column to obtain an optimal 260/230 ratio, this appeared not feasible for in particular the diaphragm samples.

Comparing results obtained with the four analyses tools, we observed differences in overall stability of the genes between them. For geNorm, BestKeeper and deltaCt, overall stability was modest, while contrastingly, NormFinder revealed high stability through the whole panel. This might result from its statistical model, with the integration of technical and biological variability, but we also found that the ranking of genes was similar to other methods, regardless of the stability. As we have noted before, use of multiple analysis methods provides more reliable data analysis, and use of large sample sets permits detection of subtle condition-specific variations. We also show that the use of two high-scoring reference genes is sufficient for normalization of qRT-PCR data since the addition of three or more genes resulted in near identical normalization factors (S6 Fig and S3 Table).

Historically, *Actb* and *Gapdh* have been prevalently used as reference genes for normalization in BL10-*mdx* mice. Over the last years, studies have shown that these genes however perform poorly, as they are not very stably expressed [36]. In our study, *Actb* was identified as highly scoring in healthy samples (regardless of the method used for analysis), but was near-unanimously ranked last when dystrophic samples were included. Our validation strategy (Fig 8B) revealed that this gene is indeed highly stable in healthy tissue, but expression is markedly elevated in dystrophic tissue, and moreover alters with increasing age, rendering it unsuitable for normalization. We also confirmed that *Gapdh* is a poor reference, ranking consistently low and exhibiting highly variable expression, even following normalization. *Rpl13a* was previously described as a suitable reference gene for the BL10-*mdx* mouse, despite modestly elevated expression in dystrophic tissue [34]. In our results, *Rpl13a* was ranked as one of the lowest scoring genes. We reveal that this gene indeed exhibits a modest increase in expression within dystrophic tissue, but in addition, skeletal muscle is markedly increased (~5-fold) at 4 weeks of age specifically. Our validation strategy established that stable genes can be correctly recognized as such: normalizing one of our stronger reference genes, *Htatsf1*, using our two strongest genes (*Pak1ip1* and *Zfp91*) demonstrated that expression of this gene does not significantly alter with age, tissue or disease (S5C–D Fig).

The results of Boccanegra *et al*. [43] were independently obtained in an tissue-, strain- and age-matched sample set. While these authors did not use an identical panel of genes, sufficient overlap exists to compare and contrast these studies. *Pak1ip1*, the strongest candidate reference gene from our panel, was of modest stability in their dataset. However, it performed better in the tissue-specific subsets, being ranked as one of the best scoring genes in diaphragm or heart only. We did not observe tissue type dependent differences as *Pak1ip1* consistently scored highly, regardless of the tissue, age or disease. Conversely, they report that *Ap3d1* was one of their strongest candidates, where in our hands the same gene was mid-ranking. It should be noted, however, that only slight differences exist between high and mid-ranking candidates in both studies (i.e., most genes are acceptable candidates, if not necessarily the strongest), while concomitantly both studies identify several genes as actively poor candidates: here stronger consensus was found. Boccanegra *et al* reached similar conclusions to ours regarding *Rpl13a* (confirming disease-associated variation in expression of this gene) and recognized *Gapdh* as a gene with tissue- and age-associated changes, in strong agreement with our data. For studies of healthy or dystrophic murine tissue, these genes should not be used as references. Interestingly, however, *Actb* was one of the favorable reference genes in the panel of Boccanegra *et al*., although this gene was found unsuitable due to high variability in our study, and also ranked poorly in a comparable study in the DE50-MD canine model of DMD [35]. As we assessed the same mouse strains, tissues and ages, but used samples from different individual mice, these discrepancies presumably reflect local differences (mouse husbandry and food), or methodological differences (primer design and quality, sample quality and preparation, reagents and instrumentation), which are more challenging to exactly replicate between independent laboratories. While these conflicting results render *Actb* contentious as a reference for muscle, the otherwise strong agreement between our studies identify both consistently strong and consistently poor candidates, and moreover highlights the value of independent, parallel studies of this nature.

## Conclusions

In this study we performed a comprehensive analysis of nine candidate reference genes across the gastrocnemius, diaphragm and heart of BL10-*mdx* and D2-*mdx* mice, and their corresponding wild types aged 4 to 52 weeks. We revealed that *Htatsf1, Pak1ip1* and *Zfp91* are suitable reference genes for the normalization of RT-qPCR data in these samples, regardless of strain, tissue type, or age. The selection of suitable reference genes is essential as stability and reliability can differ between studies differing in sample characteristics and methodology.

## Supporting information

S1 Table Average Cq values per tissue and per strain. (TIF)

S2 Table Data characteristics of the Cq values of nine candidate reference genes. Data represent all samples combined. Min, minimum; Max, maximum; SD, standard deviation.
(TIF)

S3 Table Pairwise variation (V value) of candidate reference genes analyzed using geNorm. The V2/3 refers to the pairwise variation between the normalization factors when using two reference genes compared to using three reference genes. Variation below 0.2 is considered acceptable, which was met in all conditions.
(TIF)

S1 Fig geNorm rankings. Rankings of the pairwise stability value (M) of the nine candidate reference genes, assessed by the geNorm method, for (**A**) strain-specific subsets, and (**B**) age-specific subsets. Genes are ranked from low stability (high M value) to high stability (low M value), where M values <  0.5 indicate highly stable genes. The highest scoring genes (the ‘best pair’) are considered to be equal in M value.
(TIF)

S2 Fig BestKeeper rankings. Rankings of the coefficient of correlation (r) of the nine candidate reference genes, assessed by the BestKeeper method, for (**A**) strain-specific subsets, and (**B**) age-specific subsets, including the geometric mean scores of all ages. Genes are ranked from low stability (low r) to high stability (high r).
(TIF)

S3 Fig deltaCt rankings. Rankings of the average deltaCt standard deviation of the nine candidate reference genes, assessed by the deltaCt method, for strain-specific subsets. Genes are ranked from low stability (high deltaCt score) to high stability (low deltaCt score).
(TIF)

S4 Fig NormFinder rankings (ungrouped). Ungrouped rankings of the stability value of the nine candidate reference genes, assessed by the NormFinder method, for strain-specific subsets. Genes are ranked from low stability (high stability value) to high stability (low stability value).
(TIF)

S5 Fig Normalization of Gapdh and Htatsf1. (**A**) Mean raw RQ values for Gapdh in gastrocnemius, diaphragm and heart. (**B**) Normalization to the geometric mean of *Htatsf1*, *Pak1ip1* and *Zfp91* in gastrocnemius, diaphragm and heart shows reduced variation. (**C**) Mean raw RQ values for *Htatsf1* in gastrocnemius, diaphragm and heart. (**D**) Normalization to the geometric mean of *Pak1ip1* and *Zfp91* in gastrocnemius, diaphragm and heart shows limited variation. Data is shown as log10 of RQ values. CoV values represent the average of the individual CoVs per time-point, per tissue. Data is separated by strain: Light blue: BL10-wt; Dark blue: BL10-*mdx*; Light red: D2-wt; Dark red: D2-*mdx*.
(TIF)

S6 Fig Normalization factors of high-scoring genes. Association between normalization factors from three high-scoring genes (*Htatsf1*, *Pak1ip1* and *Zfp91*) and normalization factors from two high-scoring genes (*Pak1ip1* with either (**A**) *Htatsf1* or (**B**) *Zfp91*) show largely similar gradient and Pearson r values (indicated on plots). Each datapoint represents the normalization factor of an individual sample.
(TIF)
