# Supplementary figures and images for: Identification of suitable qPCR reference genes for the normalization of gene expression in the BL10-mdx and D2-mdx mouse models of Duchenne muscular dystrophy
Source: PLoS One. 2025 Feb 25;20(2):e0318944. doi: 10.1371/journal.pone.0318944 (PMC11856590; doi:10.1371/journal.pone.0318944)

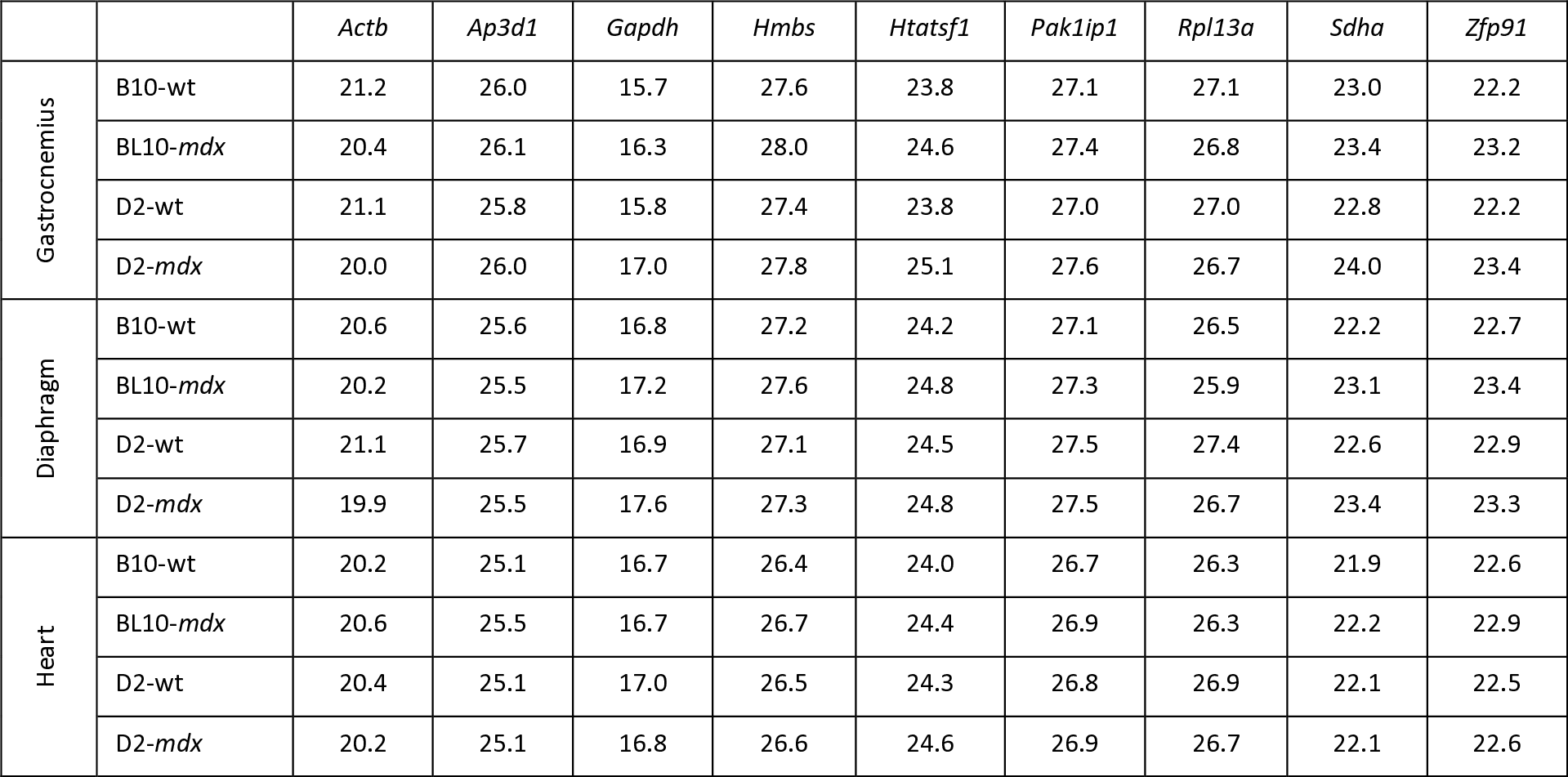

Supplement: S1 Table — (TIF) [file pone.0318944.s001.tif]

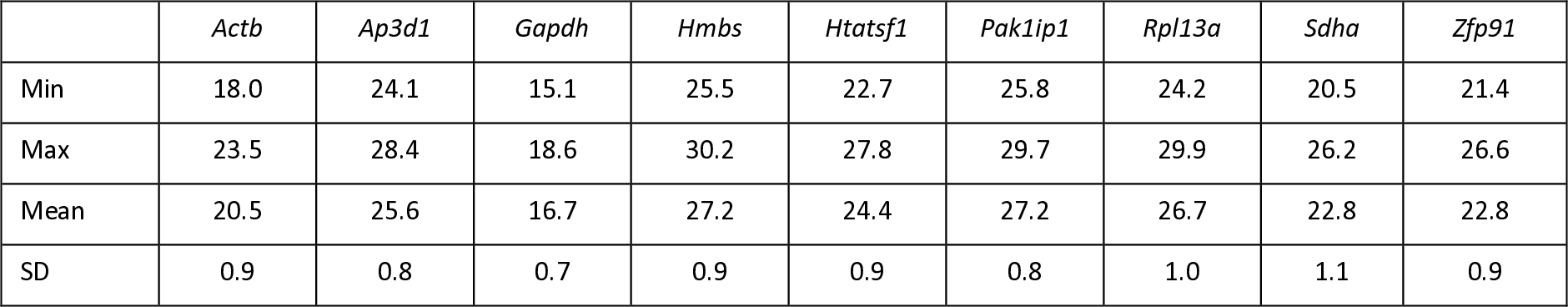

Supplement: S2 Table — Data represent all samples combined. Min, minimum; Max, maximum; SD, standard deviation. (TIF) [file pone.0318944.s002.tif]

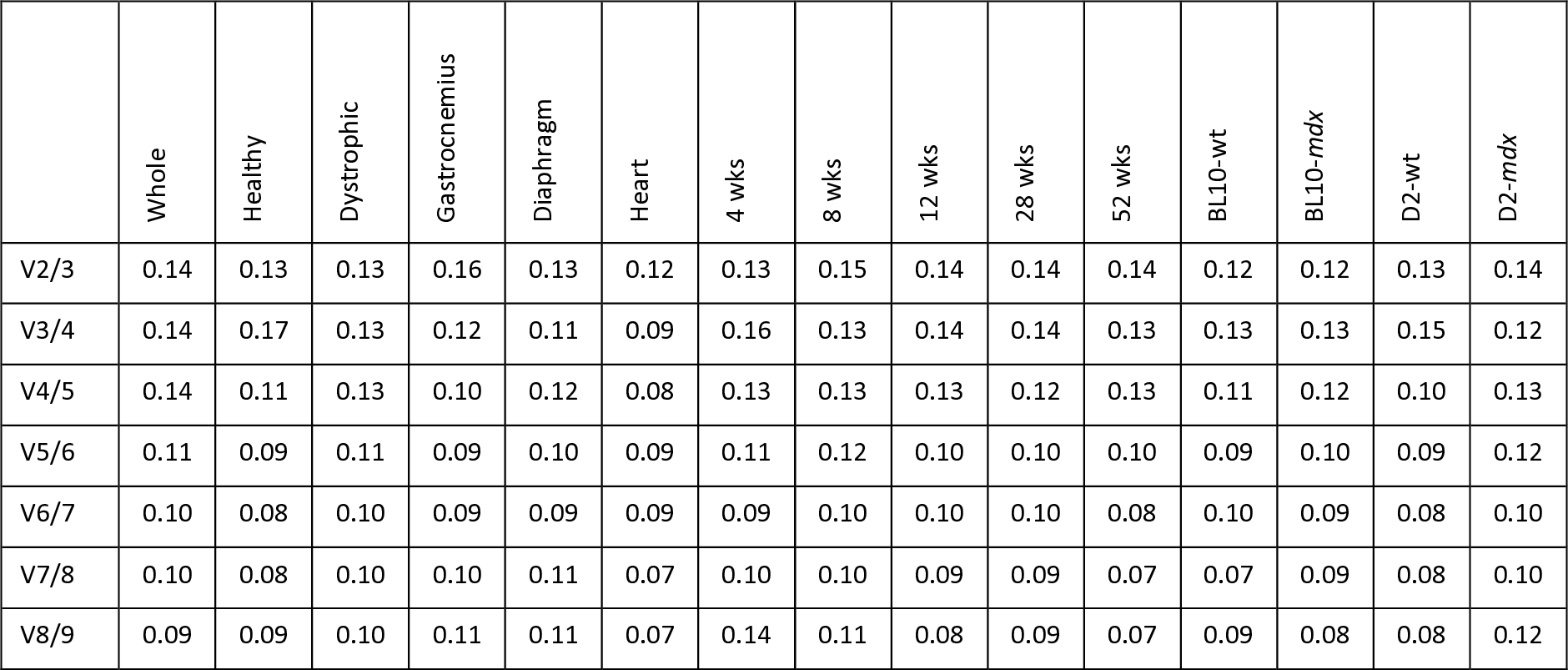

Supplement: S3 Table — The V2/3 refers to the pairwise variation between the normalization factors when using two reference genes compared to using three reference genes. Variation below 0.2 is considered acceptable, which was met in all conditions. (TIF) [file pone.0318944.s003.tif]

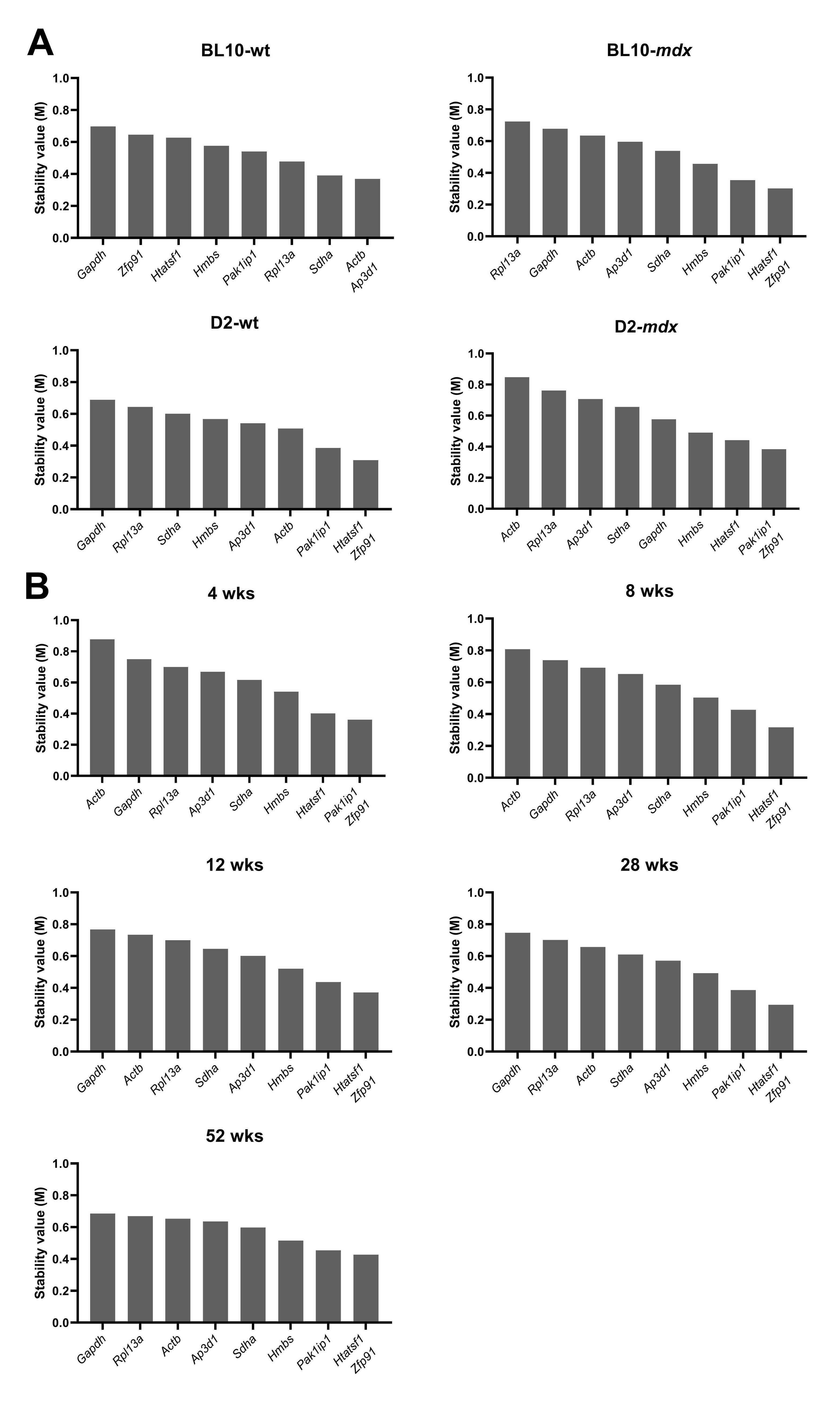

Supplement: S1 Fig — Rankings of the pairwise stability value (M) of the nine candidate reference genes, assessed by the geNorm method, for (A) strain-specific subsets, and (B) age-specific subsets. Genes are ranked from low stability (high M value) to high stability (low M value), where M values < 0.5 indicate highly stable genes. The highest scoring genes (the ‘best pair’) are considered to be equal in M value. (TIF) [file pone.0318944.s004.tif]

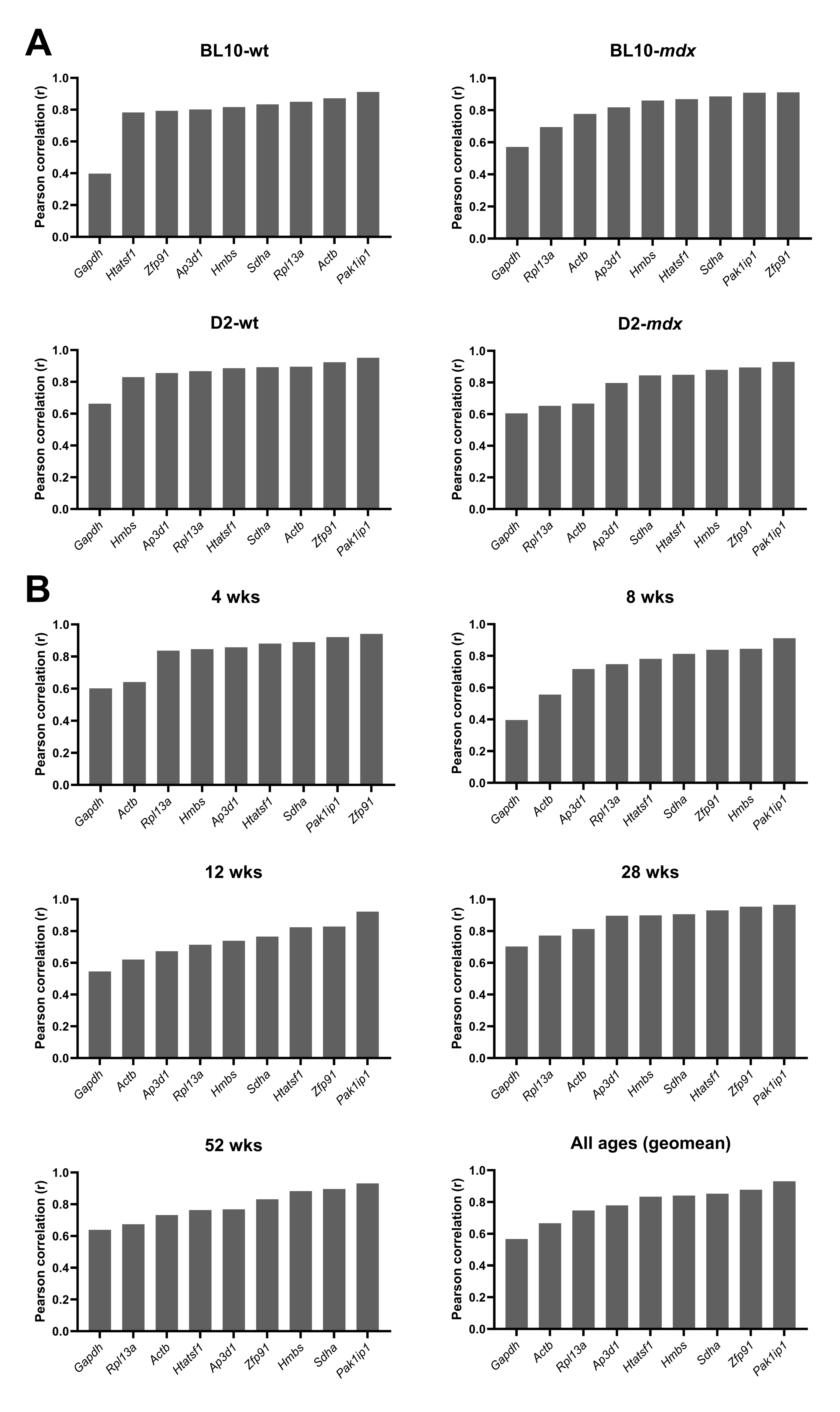

Supplement: S2 Fig — Rankings of the coefficient of correlation (r) of the nine candidate reference genes, assessed by the BestKeeper method, for (A) strain-specific subsets, and (B) age-specific subsets, including the geometric mean scores of all ages. Genes are ranked from low stability (low r) to high stability (high r). (TIF) [file pone.0318944.s005.tif]

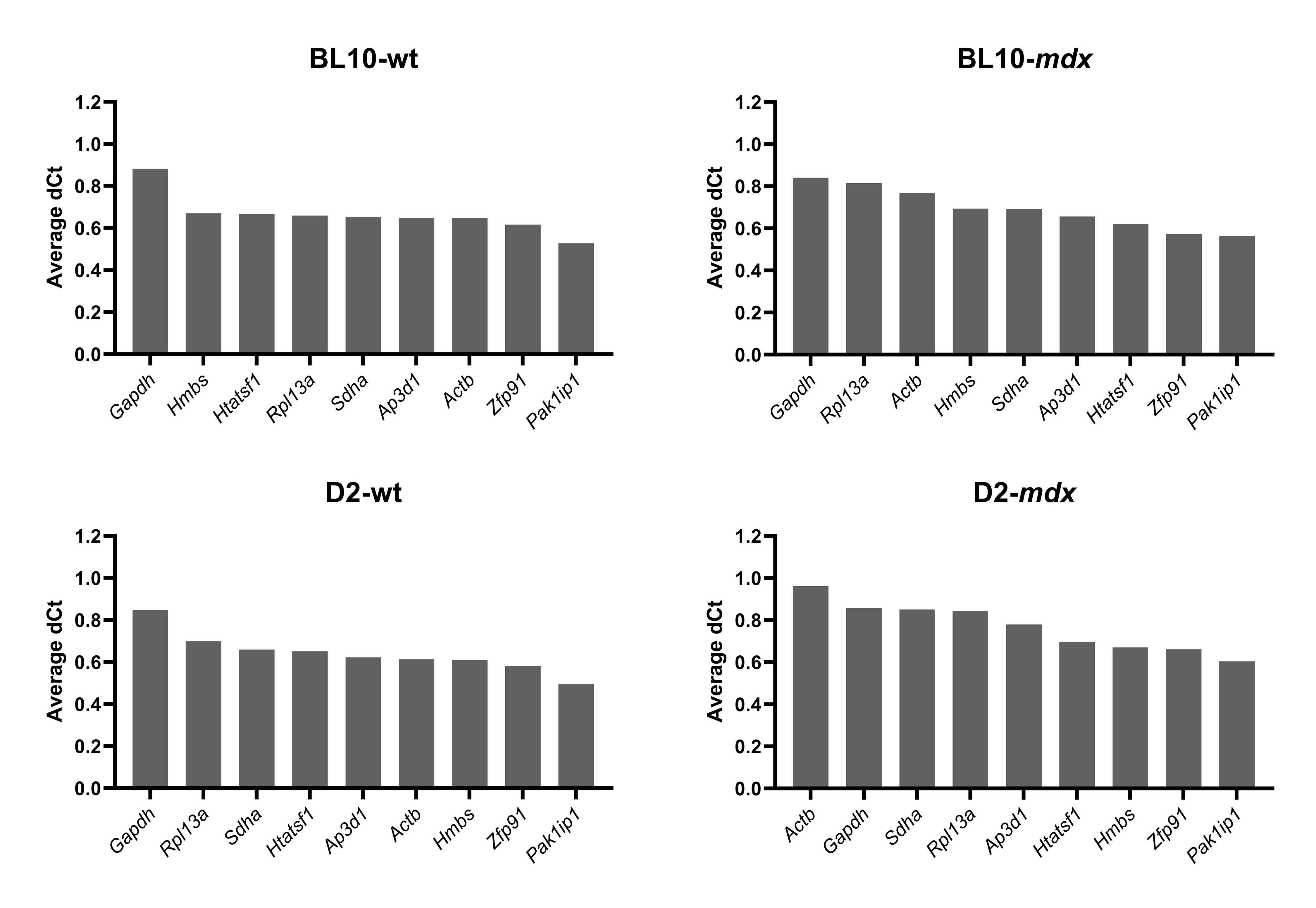

Supplement: S3 Fig — Rankings of the average deltaCt standard deviation of the nine candidate reference genes, assessed by the deltaCt method, for strain-specific subsets. Genes are ranked from low stability (high deltaCt score) to high stability (low deltaCt score). (TIF) [file pone.0318944.s006.tif]

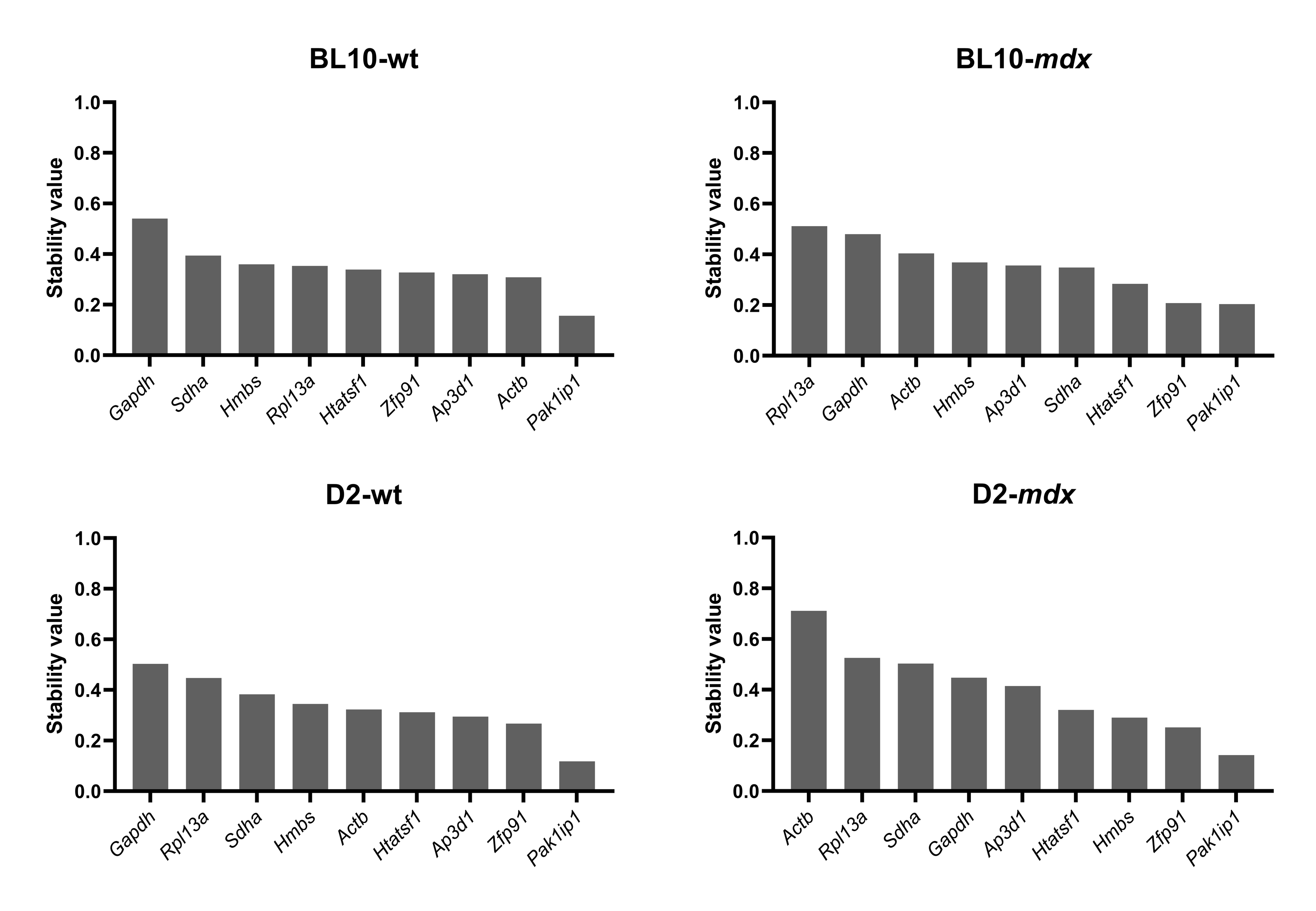

Supplement: S4 Fig — Ungrouped rankings of the stability value of the nine candidate reference genes, assessed by the NormFinder method, for strain-specific subsets. Genes are ranked from low stability (high stability value) to high stability (low stability value). (TIF) [file pone.0318944.s007.tif]

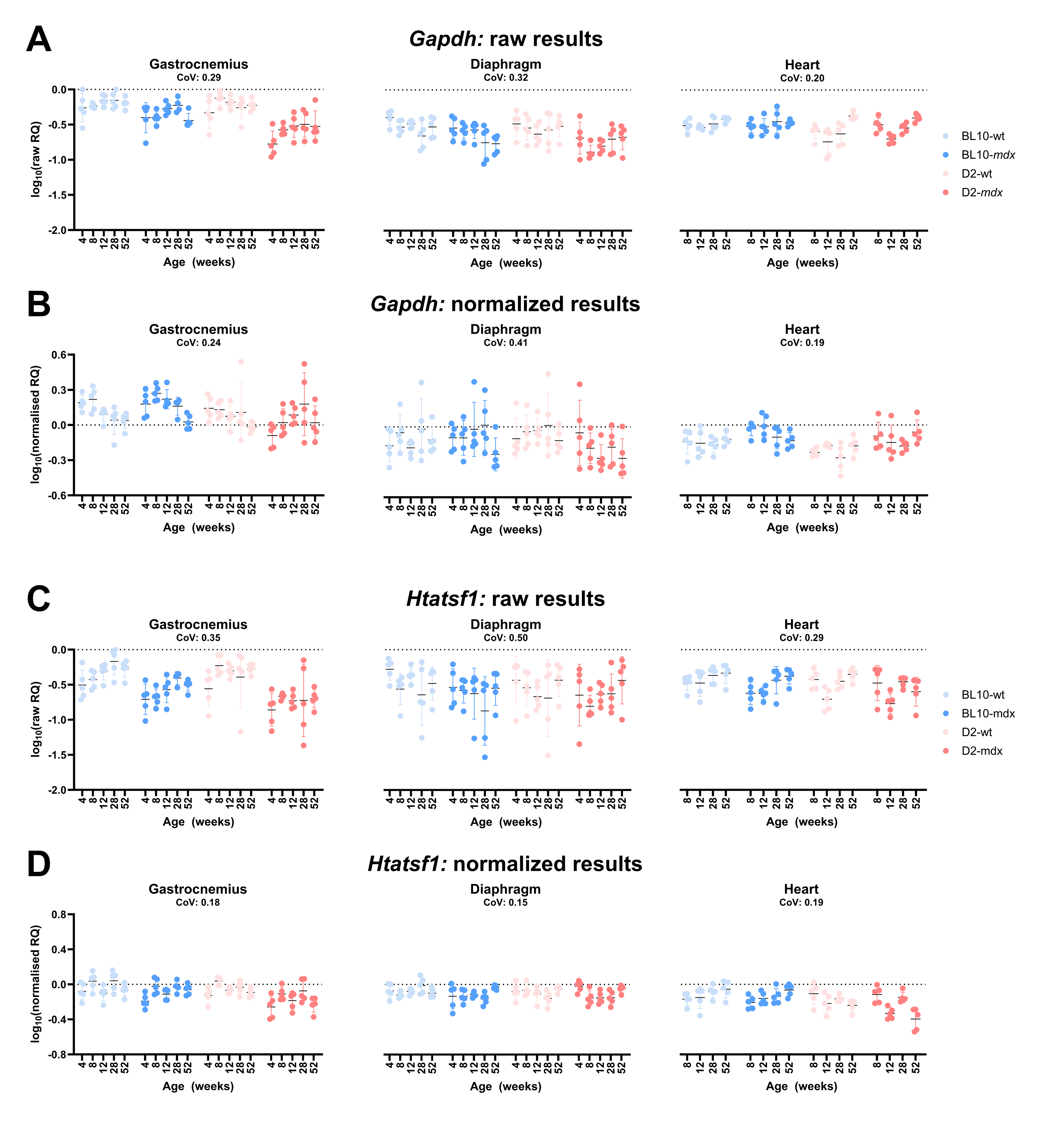

Supplement: S5 Fig — (A) Mean raw RQ values for Gapdh in gastrocnemius, diaphragm and heart. (B) Normalization to the geometric mean of Htatsf1, Pak1ip1 and Zfp91 in gastrocnemius, diaphragm and heart shows reduced variation. (C) Mean raw RQ values for Htatsf1 in gastrocnemius, diaphragm and heart. (D) Normalization to the geometric mean of Pak1ip1 and Zfp91 in gastrocnemius, diaphragm and heart shows limited variation. Data is shown as log10 of RQ values. CoV values represent the average of the individual CoVs per time-point, per tissue. Data is separated by strain: Light blue: BL10-wt; Dark blue: BL10-mdx; Light red: D2-wt; Dark red: D2-mdx. (TIF) [file pone.0318944.s008.tif]

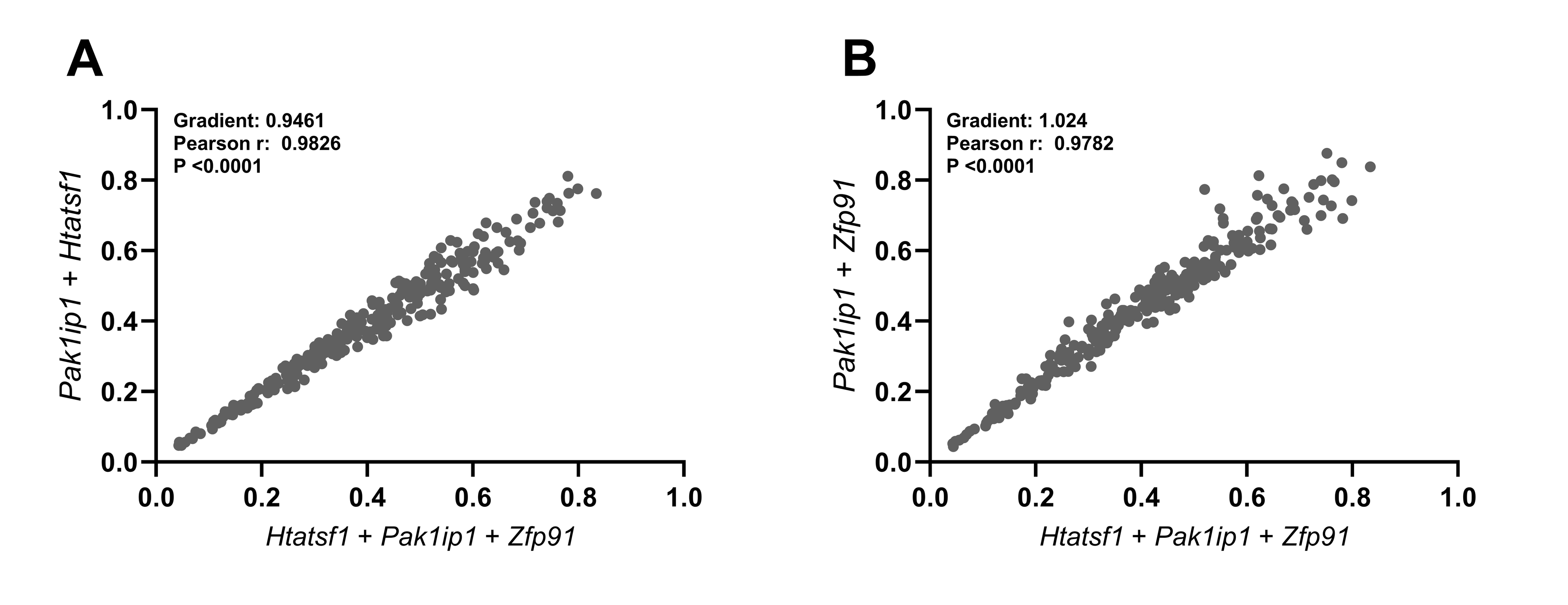

Supplement: S6 Fig — Association between normalization factors from three high-scoring genes (Htatsf1, Pak1ip1 and Zfp91) and normalization factors from two high-scoring genes (Pak1ip1 with either (A) Htatsf1 or (B) Zfp91) show largely similar gradient and Pearson r values (indicated on plots). Each datapoint represents the normalization factor of an individual sample. (TIF) [file pone.0318944.s009.tif]
